# Metal and Silicate Particles Including Nanoparticles Are Present in Electronic Cigarette Cartomizer Fluid and Aerosol

**DOI:** 10.1371/journal.pone.0057987

**Published:** 2013-03-20

**Authors:** Monique Williams, Amanda Villarreal, Krassimir Bozhilov, Sabrina Lin, Prue Talbot

**Affiliations:** 1 Department of Cell Biology and Neuroscience, University of California Riverside, Riverside, California, United States of America; 2 Central Facility for Advanced Microscopy and Microanalysis, University of California Riverside, Riverside, California, United States of America; University of Kansas, United States of America

## Abstract

**Background:**

Electronic cigarettes (EC) deliver aerosol by heating fluid containing nicotine. Cartomizer EC combine the fluid chamber and heating element in a single unit. Because EC do not burn tobacco, they may be safer than conventional cigarettes. Their use is rapidly increasing worldwide with little prior testing of their aerosol.

**Objectives:**

We tested the hypothesis that EC aerosol contains metals derived from various components in EC.

**Methods:**

Cartomizer contents and aerosols were analyzed using light and electron microscopy, cytotoxicity testing, x-ray microanalysis, particle counting, and inductively coupled plasma optical emission spectrometry.

**Results:**

The filament, a nickel-chromium wire, was coupled to a thicker copper wire coated with silver. The silver coating was sometimes missing. Four tin solder joints attached the wires to each other and coupled the copper/silver wire to the air tube and mouthpiece. All cartomizers had evidence of use before packaging (burn spots on the fibers and electrophoretic movement of fluid in the fibers). Fibers in two cartomizers had green deposits that contained copper. Centrifugation of the fibers produced large pellets containing tin. Tin particles and tin whiskers were identified in cartridge fluid and outer fibers. Cartomizer fluid with tin particles was cytotoxic in assays using human pulmonary fibroblasts. The aerosol contained particles >1 µm comprised of tin, silver, iron, nickel, aluminum, and silicate and nanoparticles (<100 nm) of tin, chromium and nickel. The concentrations of nine of eleven elements in EC aerosol were higher than or equal to the corresponding concentrations in conventional cigarette smoke. Many of the elements identified in EC aerosol are known to cause respiratory distress and disease.

**Conclusions:**

The presence of metal and silicate particles in cartomizer aerosol demonstrates the need for improved quality control in EC design and manufacture and studies on how EC aerosol impacts the health of users and bystanders.

## Introduction

Electronic cigarettes (EC) are generally manufactured in China and are rapidly gaining acceptance in many countries [1], [2]. In the United States, EC are available on the Internet, in malls, and in local shops. They have become an integral part of the environment without much information regarding the quality control used in their manufacture or their health effects [3].

EC deliver aerosolized nicotine to users and may serve as a surrogate for conventional tobacco-containing cigarettes [4], [5]. Puffing an EC activates a battery that in turn heats liquid containing flavoring, a humectant(s) such as propylene glycol or vegetable glycerin, and nicotine. Some models, such as the one used in this study, do not contain nicotine. Early models of EC had separate atomizers for heating and cartridges for holding fluid [3]. As EC have evolved, the atomizer and cartridge have often been combined into a single unit called a “cartomizer” [6]. Recent studies have shown that EC can deliver nicotine to users, although not always as effectively as conventional cigarettes [7], [8].

EC may help smokers overcome nicotine addiction and/or serve as nicotine delivery devices that are safer than tobacco burning cigarettes [9], [10]. EC have helped some smokers stop using conventional brands and either quit smoking entirely or switch to the presumably safer EC [11], [12]. Their effectiveness in this regard may be aided by the hand-mouth motion that the EC provides, unlike nicotine patches and gum. Some users have reported to us that EC have helped them break nicotine addiction, but not the hand-mouth addiction associated with smoking.

Because EC do not burn tobacco, they do not produce the numerous chemicals found in conventional tobacco smoke. For this reason, they may be safer than conventional cigarettes [5], although the short and long-term health effects of EC are just beginning to be understood and some serious complications associated with EC use have recently been reported [13], [14]. Concerns about quality control in the manufacture of these products have been raised, and packaging, labeling, and poor quality control are some of the issues that need to be addressed [3]. In addition, the Food and Drug Administration (FDA) found diethylene glycol, a toxic chemical that can cause death, in one EC cartridge [15], and some bottles of EC refill fluid labeled “no nicotine” did in fact contain significant amounts of nicotine [16]. Product performance is often highly variable among brands and even variable within the same model of a particular brand [6], [17], [18]. The contents and aerosol production of EC cartomizers have not received much prior evaluation. Because EC contain various metal components, we hypothesized that their aerosol would contain metals. The purpose of this study was to test this hypothesis by analyzing the structural and elemental contents, cytotoxicity, and aerosol emissions of cartomizers from a leading brand of EC.

## Results

### Cartomizer dissections

**Figure 1:**
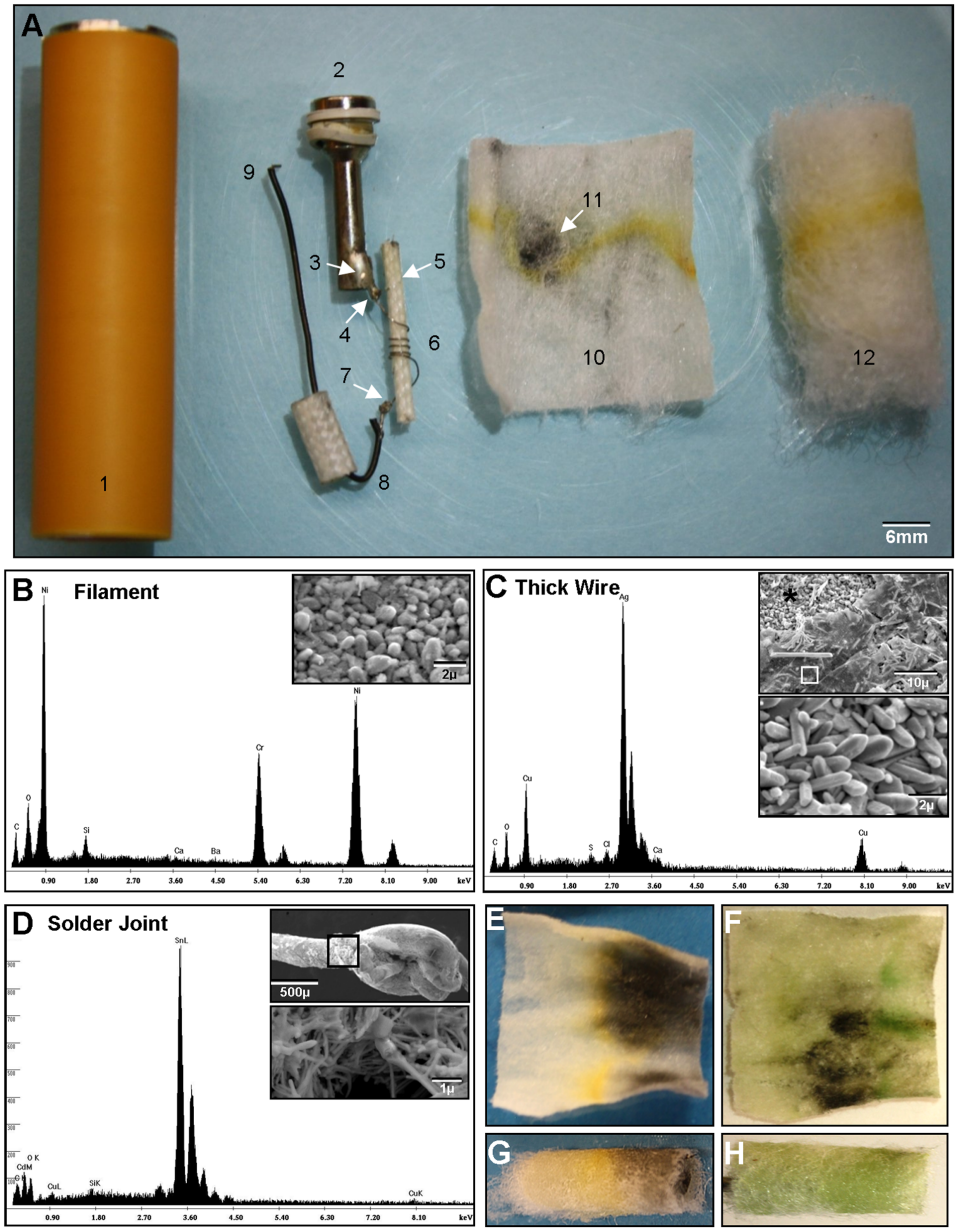
Cartomizer anatomy. (A) A dissected cartomizer. 1 = mouthpiece, 2 = air tube, 3 = solder joint between air tube and thick wire, 4 = solder joint between thick wire and filament, 5 = wick, 6 = filament, 7 = solder joint between the filament and thick wire, 8 = thick wire, 9 = solder joint where the thick wire would attach to the mouthpiece, 10 = inner fibers, 11 = black area on inner fibers, 12 = outer fibers with yellow electrophoretic band. (B) EDS spectrum showing that the filament is comprised of chromium and nickel. Insert shows the particulate surface of the filament. (C) Scanning electron micrographs (inserts) and EDS spectrum of the thick wire which is comprised of copper coated with silver. Asterisks (*) in the upper insert indicates area where silver coating is missing and copper wire is exposed; white box indicates the silver coating. Lower insert shows the surface of the copper wire at high magnification. (D) EDS spectrum showing that solder joints are comprised mainly of tin. Upper insert shows a typical poor quality solder joint between the filament and thick wire. Boxed area is shown at higher magnification in the lower insert and contains tin whiskers. (E–H) Images of fiber types, (E, F) inner fibers, and (G, H) outer fibers. (E, G) black deposits on fibers and (F, H) green coloration on both sets of fibers.

Twenty-two fresh cartomizers from a leading manufacturer were dissected and analyzed using scanning electron microscopy (SEM) and electron dispersion spectroscopy (EDS) (Figure 1A). All cartomizers consisted of a cylindrical sheath or mouthpiece, a wick, two thick and one thin (filament) wire, an air tube, four solder joints, inner dense fibers, and outer fibers, which appeared to be Poly-fil (Figure 1A). The filament (thin wire) was wound around the wick, which appeared to be fiberglass (Figure S1A). The wick was fully dissected from 13 cartomizers, and in 12 of these, black debris was present on the wick (not shown). The surface of the filament was comprised of numerous small particles containing nickel and chromium (nichrome) (Figures 1B and insert). The thick wire was made of copper coated with silver (Figures 1C and inserts). In some areas, silver was missing, and copper could be viewed directly (Figure 1 C asterisks in upper insert). The surface of the copper wire, like the nichrome wire, was comprised of many small particles (Figure 1C lower insert). The ends of the filament wound around and were soldered to one end of each thick wire (Figure 1A, D). The solder joints contained mainly tin plus a small amount of copper, and were usually poorly formed (Figure 1 D insert). Tin “whiskers” (microscopic, conductive, crystals that emanate spontaneously from pure tin) were present on solder joints and on the wires near joints (Figure 1 D and insert). The opposite end of each thick wire was soldered to either the inside of the mouthpiece or the air tube (Figure 1A). The mouthpiece was comprised primarily of iron, chromium, and manganese, which are characteristic of stainless steel (Figure S1B), and the air tube was made of nickel (Figure S1C). The white gasket at the end of the air tube was comprised mainly of silica (Figure S1D).

### Inner and outer fibers

In 21 of 22 cartomizers, the inner fibers had black debris and burn spots near the filament (Figures 1 A, E, F), and in 2 of 22 cartomizers, the inner fibers were green (Figure 1F). 10 of 22 outer fibers had black deposits (Figure 1G), and the outer fibers in two cartomizers were green (Figure 1H). All cartomizers showed apparent “electrophoretic” movement of cartomizer fluid toward the battery end (Figure 1A).

### Centrifugation of the outer and inner fibers

**Figure 2:**
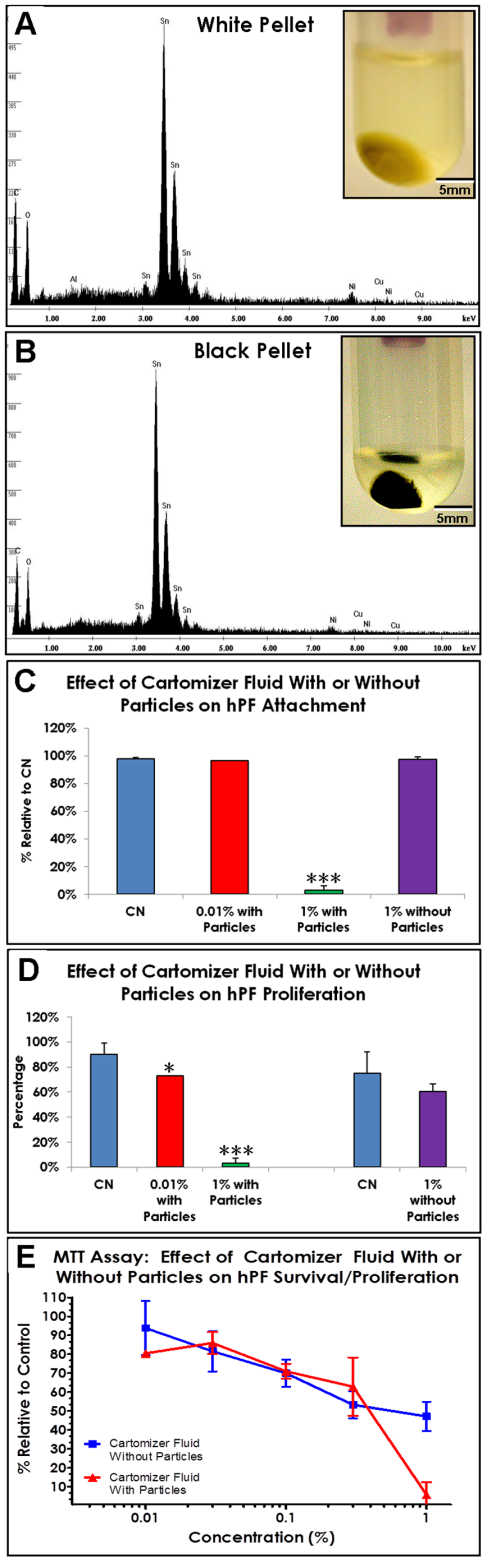
Elemental analysis of pellets, cartomizer fluid, and particles on fibers. (A and B) Supernatants and pellets obtained by centrifuging outer fibers (A) and inner fibers (B) (inserts). EDS spectra showing that the pellets are comprised mainly of tin. (C) Cartomizer fluid with particles inhibited hPF attachment dose dependently. (D) Cartomizer fluid with particles inhibited proliferation dose dependently. (E).Cartomizer fluid with tin particles had a stronger negative effect on hPF survival in the MTT assay than fluid without particles.

The outer and inner fibers were centrifuged either together (5/7) or separately (2/7). All cartomizers that were centrifuged produced a sizable pellet (Figures 2A and B inserts). When both fiber types were centrifuged together, a layered black-yellow-white pellet formed (not shown). However, centrifugation of the outer and inner fibers separately produced yellow-white pellets (Figure 2A insert) and black pellets (Figure 2 B insert), respectively. The means ± standard deviations for the wet and dry weights of the pellets were: white pellet wet = 50 mg±30 mg and dry = 20 mg±40 mg; black pellet wet = 6 mg±6 mg and dry = 0.2 mg±0.2 mg. Both the white and black pellets obtained by centrifugation were analyzed using EDS microanalysis in the SEM and contained tin particles (Figures 2 A, B).

### Cytotoxicity of cartomizer fluid and pellets when tested with human pulmonary fibroblasts (hPF)

The cytotoxicity of cartomizer fluids with and without tin particles was evaluated using live cell imaging and the MTT assay (Figure 2). Cartomizer fluid with tin particles inhibited both attachment (Figure 2C) and proliferation (Figure 2D) dose dependently, while cartomizer fluid without tin did not significantly affect either of these processes (Figures 2C, D). In the MTT assay, fluid with and without particles inhibited hPF survival at a dose of 1%, and this effect was strongest when particles were present (Figure 2E).

### Distribution of tin in cartomizer fluid and fibers

**Figure 3:**
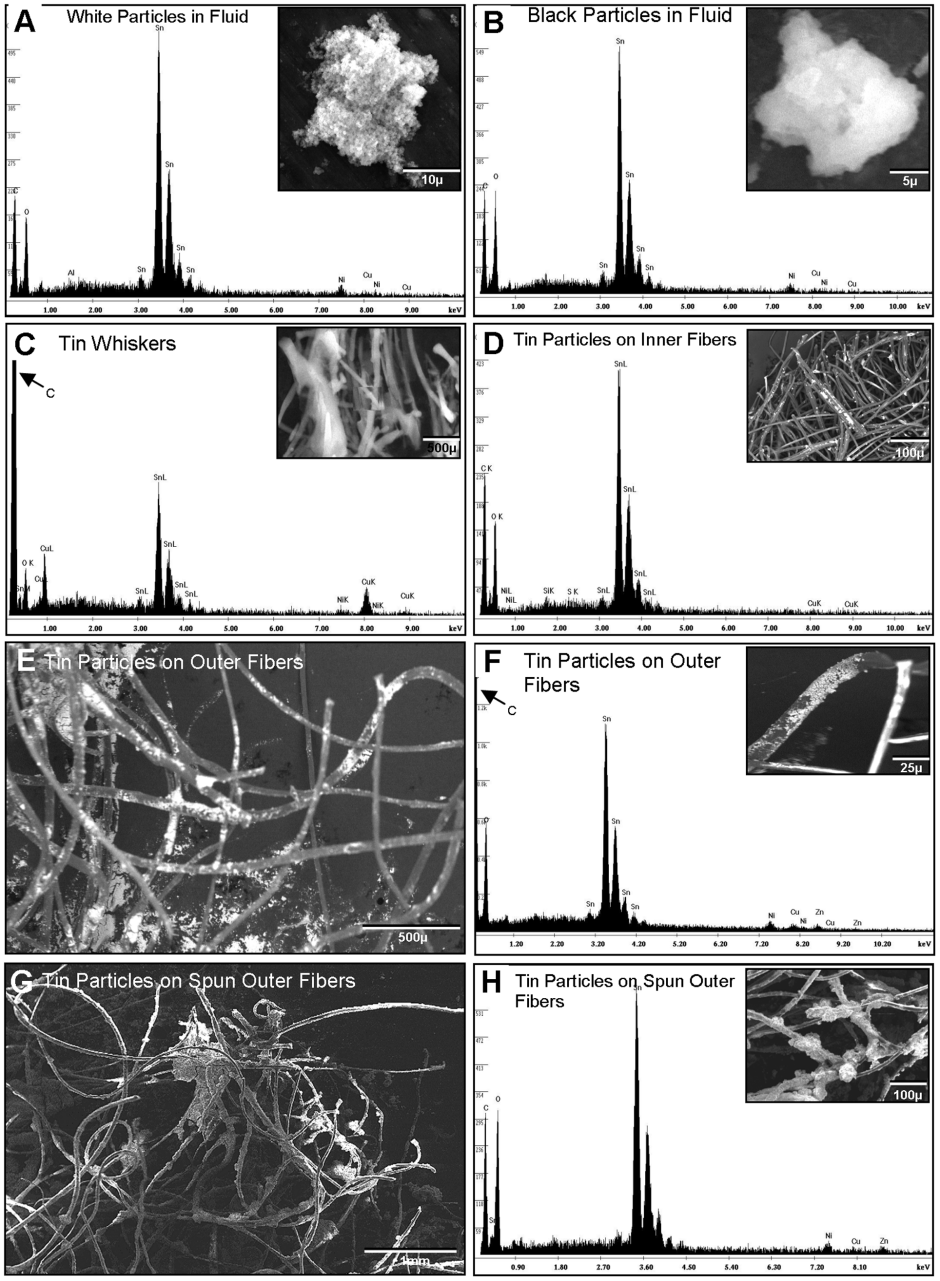
EDS spectra of elemental analysis and SEM images of cartomizer fluid and particles on fibers. (A–B) Micrographs of white and black particles from cartomizer fluid (inserts) and spectra showing that the particles in fluid contained mainly tin. (C) Spectrum and micrograph (insert) of tin whiskers found in cartomizer fluid. (D) Spectrum and micrograph (insert) of inner fibers showing that the particulate deposition on the fibers is mainly tin. (E) Green outer fibers showing particles coating the fibers. (F) Micrograph (insert) and spectrum of green coated outer fibers. Particles are mainly tin with a trace of copper. (G) Green outer fibers after centrifugation, which removed green coloration. (H) Micrograph (insert) and spectrum of centrifuged outer fibers at higher magnification. Particles on the surface of the fibers are mainly tin, without copper.

To determine where tin was distributed in the cartomizers, the fluid and fibers were examined in the SEM (Figure 3). Cartomizer fluid from the inner and outer fibers contained white and black particles that were shown by EDS microanalysis to be tin (Figures 3 A, B). Tin whiskers were also present in fluid that came off the fibers (Figure 3 C). Particles and whiskers contained mainly tin with minor amounts of copper and nickel. The surfaces of the inner fibers had particles, which were also tin (Figure 3 D). Backscatter imaging in the SEM of green outer fibers, which had not been centrifuged, revealed that they were coated with numerous bright particles indicative of heavy elements (Figure 3 E). These particles were mainly tin with traces of copper (Figure 3 F). When these outer fibers were centrifuged, most of the green coloration was removed, and the fibers were coated with tin particles (Figure 2G) containing very little copper (Figure 2H).

### Analysis of particles in aerosol

**Figure 4:**
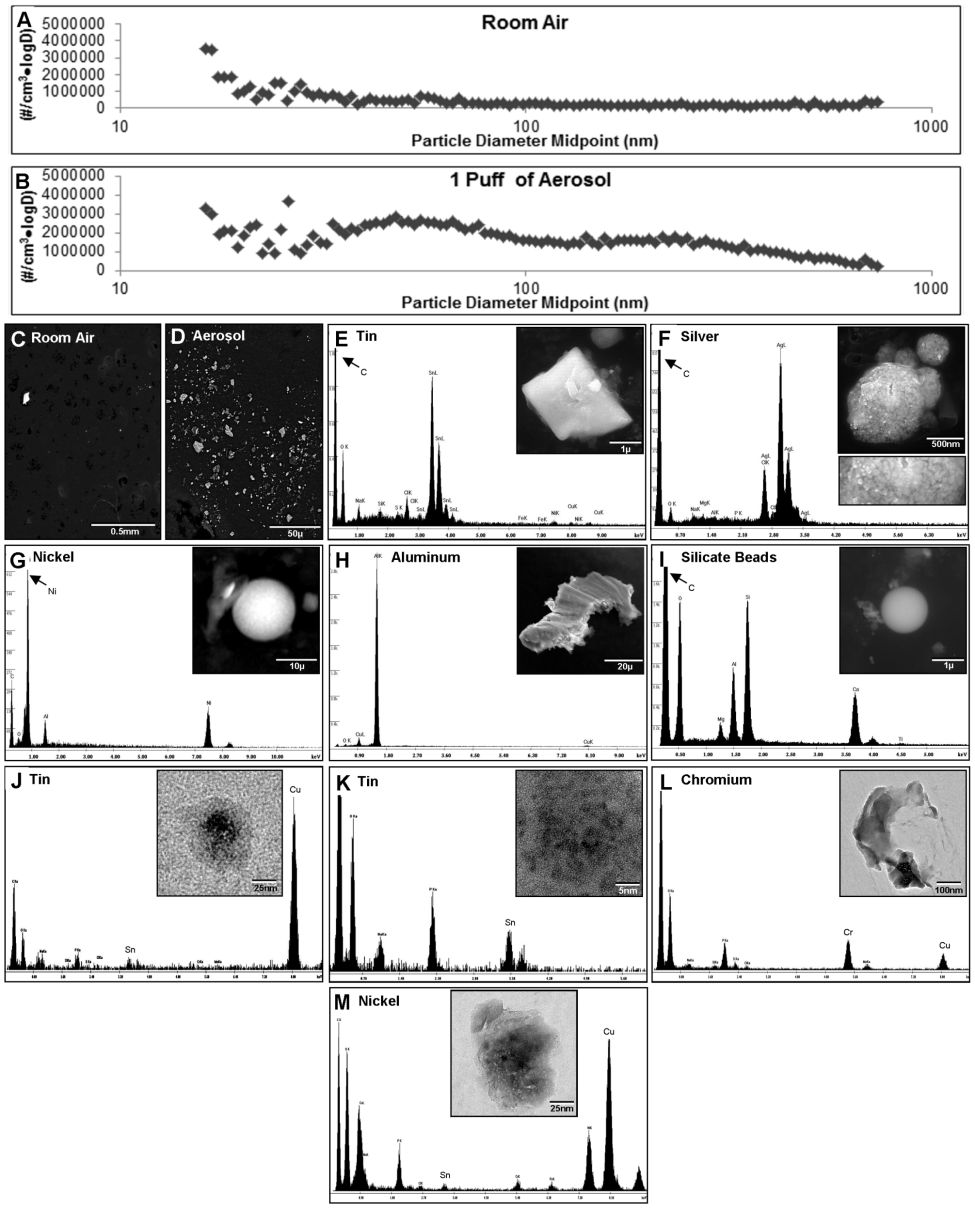
Particulate material in the aerosol: Size distribution of particles in room air (A) and in aerosol (B) is expressed as number of particles/(cm3*logD). The number of particles/cm3 with diameters between logD1 and logD2 is equal to the area under the curve bounded by logD1 and logD2. (C–D) Low magnification SEM micrographs of particles from room air (C) and aerosol (D) viewed in the backscatter mode. (E–I) EDS spectra and SEM micrographs (inserts) of particles in aerosol. F (insert) shows aggregation of small silver particles. (J–M) EDS spectra and TEM micrographs (inserts) of tin (J–K), chromium (L), and nickel (M) nanoparticles in aerosol.

The size distribution of particles less than 1000 nm in room air and in one puff of cartomizer aerosol were compared (Figures 4 A, B). Room air contained relatively few particles, most of which were at the small end of the size range (Figure 4A). In contrast, one puff of cartomizer aerosol contained numerous particles distributed in two very broad peaks. Most of these particles were below 100 nm in diameter (Figure 4 B).

Particles collected from room air and cartomizer aerosol were next analyzed with SEM and EDS microanalysis (Figures 4 C–I). Using the compositional contrast backscatter-imaging mode in the SEM, room air samples had few bright particles indicating few heavy elements (Figure 4 C), in contrast to aerosol samples, which had numerous bright particles (Figure 4D). Particles in room air were mainly calcium, potassium, silicon, aluminum, and sodium (not shown). Further EDS elemental analysis of the bright particles in aerosol revealed they contained mainly tin (Figure 4 E), silver (Figure 4 F), nickel (Figure 4 G), and aluminum (Figure 4 H). Particles of iron, cerium, lanthanum, bismuth, and zinc were also found in the SEM analysis (not shown). Numerous round particles of various sizes that were less bright in the backscatter electron mode contained mainly silicon with lesser amounts of magnesium, aluminum and calcium (Figure 4 I).

Particles analyzed in the SEM ranged in size from about 1 to 20 µm. To determine if metal nanoparticles (<100 nm) were present in aerosol, samples were examined by transmission electron microscopy (TEM) and EDS. Tin, chromium, and nickel nanoparticles were found in cartomizer aerosol (Figures 4 J–M). Nanoparticles of these elements were not found in samples of room air.

To quantify the abundance of specific elements in cartomizer aerosol, samples were examined using induced coupled plasma-optical emissions spectroscopy (ICP-OES). Table 1 compares the concentrations of various elements in 10 puffs of EC aerosol vs the concentrations in mainstream smoke from one cigarette (which would be approximately equivalent to 10 puffs). For 11 elements where conventional smoke data were found, the concentration of elements in EC aerosol was higher for four elements (sodium, iron, aluminum, nickel), within the conventional smoke range for five elements (copper, magnesium, lead, chromium, manganese), and lower than the range for two elements (potassium, zinc). Six elements (boron, potassium, lead, zirconium, strontium, and lithium) identified by ICP-OES were not found by EDS analysis, probably because EDS examines a small fraction of each aerosol sample. All of the elements in EC aerosol can adversely affect the respiratory system, while some also affect reproduction and development and some are carcinogens (Table 1). Silicon, calcium, aluminum, and magnesium were among the most abundant elements in aerosol, and these were found in the silicate beads and fiberglass wick during analysis by SEM and EDS.

**Table 1:** Elemental abundance in EC aerosol and cigarettes and associated health effects.

Element	Aerosol µg/10 puffs	Smoke µg/cig (∼10 puffs)	Health Effects
Sodium	4.18	1.3 [40]	Inhalation may cause lung irritation, shortness of breath, bronchitis [41].
Boron	3.83		Inhalation exposure: acute respiratory and ocular irritation [42].
Silicon	2.24		Upper respiratory irritation, coughing, shortness of breath, bronchitis [43], [44].
Calcium	1.03		Nose/throat irritation, coughing/wheezing [45].
Iron	0.52	0.042 [40]	Respiratory irritation, fume metal fever, siderosis, fibrosis [46].
Aluminum	0.394	0.22 [40]	Impaired lung function, asthma, and pulmonary fibrosis [47].
Potassium	0.292	70 [40]	May originate from silicate beads along with sodium, calcium, and magnesium.
Sulfur	0.221		Nose/throat/lung irritation, coughing, shortness of breath, and bronchitis [48].
Copper	0.203	0.19 [40]	Respiratory irritation, coughing, sneezing, thoracic pain, runny nose and vineyard sprayer's lung [49].
Magnesium	0.066	0.070 [40]	Metal fume fever, respiratory irritation, tightness in chest, difficulty breathing [50].
Zinc	0.058	0.12–1.21 [40]11.9 [51]	Metal fume fever, impaired pulmonary function, chest pain, coughing, dyspnea, shortness of breath [52].
Tin	0.037		Inorganic tin: pneumoconiosis (stannosis) and inflammation [53].
Lead	0.017	0.017–0.98 [40]0.072 [54]0.14 [51]	Can damage nervous system and kidneys [55]. Is a CA, RT, and RDT [56].
Barium	0.012		Benign pneumoconiosis [57].
Zirconium	0.007		Respiratory irritation [58].
Chromium	0.007	0.004–0.069 [40]0.0002–0.5 [54]0.0006–0.0025 [59]	Inhalation can cause respiratory irritation. Cr (VI) = carcinogen [60], [61]. Is a CA, RT, and RDT [56].
Strontium	0.006		Stable: no harmful effects at levels typically found in environment; can cause anaphylactic reaction. Radioactive: leukemia, carcinogen [62].
Nickel	0.005	0.000073 [51]0.0014–0.003 [59]	Chronic bronchitis, reduced lung function, lung inflammation, lung/nasal sinus cancer, and pulmonary fibrosis [63]. Is a CA and RT [56].
Manganese	0.002	0.003 [40]	Lung irritation, coughing, bronchitis, pneumonitis, reduction in lung function, and pneumonia [64].
Titanium	0.002		Nose/throat/lung irritation, coughing, shortness of breath, and bronchitis [65].
Lithium^a^	0.008		Nose/throat/lung irritation, coughing, shortness of breath [66].

Abbreviations: EC, electronic cigarette; CA, carcinogen; RT, respiratory toxicant; RDT, reproductive and developmental toxicant; ND, not detected.

a This value was computed using only two samples instead of three.

## Discussion

Cartomizers purchased from one manufacturer on four different occasions over a two year period showed evidence of use prior to packaging. This included apparent electrophoretic movement of the cartomizer fluid towards the battery, deposition of tin particles on the inner and outer fibers, and burning of the inner fibers. Cartomizers may have been used by the manufacturer to confirm that they produced aerosol. In some cartomizers, presale testing appeared to have been extensive (Figures 1 E, G).

Our data support the hypothesis that EC aerosol contains metal particles and are the first to report heavy metals and silicate beads in EC aerosol. In our study, centrifugation of outer and inner fibers produced large pellets of white and black tin, respectively. It is likely that the black pellets contained tin oxide, which is generally black [19], [20]. Tin oxide can be produced by heating [20], which is consistent with the black tin being located close to the filament. The green coloration observed in some cartomizer fibers may have been due to copper that migrated from the solder and/or the large wire (bare expanses of copper wire were observed using SEM - Figure 1C upper insert). Cartomizer fluid with tin particles was more cytotoxic than fluid that lacked particles when tested in vitro with hPF, suggesting that removal of tin particles from EC products would benefit the health of users. However, other factors may also affect EC cytotoxicity. A recent study showed that some brands of EC refill fluids, which do not contain tin pellets, are highly cytotoxic when tested *in vitro* with human embryonic stem cells and hPF [21].

Tin in the centrifuged pellets likely came from the solder joints or from solder that escaped into the cartomizers during manufacture or pre-sale testing. In general, solder joints were poorly formed, often appeared to be cold joints, and had rough surfaces and associated tin whiskers. No lead was found in the solder joints, which is consistent with China's ban on the use of lead in solder [22]. Lead-free solders are difficult to use and because of their high rigidity may be more fragile than solders containing lead [22]. When subjected to temperature cycling, lead containing-solders outperformed lead-free solders [22], which may be a factor in ECs undergoing cyclic temperature changes. Lead free-solders may also form tin whiskers, as was seen in EC solder joints and among cartomizer fibers. Tin whisker growth can be accelerated by electrical current at room temperature [23], and the use of the cartomizers before packaging may have facilitated tin whisker production.

The outer and inner fibers trapped many of the tin particles in the cartomizers. However, small particles comprised of various elements (tin, other metals, semimetals, and silicates) passed through cartomizer fibers and were present in aerosols. Nickel particles likely originated from the nichrome wire, and some particles in the wire had a spherical shape and size similar to the nickel particles observed in the aerosol with SEM. Silver particles may have come from the silver coating on the copper wire, as patches of silver were sometimes missing from the wire's surface. Iron was probably from other parts of the cartomizer such as the mouthpiece and/or metallic base at the battery interface. The presence of metals in EC aerosol could explain why some EC users have reported a metallic taste in their mouths when puffing (http://www.e-cigarette-forum.com/forum/health-safety-e-smoking/).

The silicate beads appeared to originate in the fiberglass wick, which had small round particles on its surface similar in size, appearance, and elemental composition to the silicate beads in the aerosol. The elements in the silicate particles (silicon, calcium, aluminum, magnesium) were among the most abundant found in the quantitative elemental analysis, suggesting that the round, smooth surfaced silicate beads pass through the outer fibers and enter aerosol more readily than the irregularly shaped and heavier tin particles, which were largely trapped among the fibers. The fiberglass wicks may be breaking down and releasing beads due to heating and the high airflow rates required to operate this brand of EC may readily carry silicate beads into the aerosol. Boron, which is used in glass manufacturing [24], was very high in concentration in the aerosol and may also have originated in the wick.

Particles larger than 1 µm and nanoparticles (16–100 nm in diameter) were present in cartomizer aerosol. The distribution of number of particles/(cm^3^*logD) is shown in Figure 4. One puff of aerosol contained approximately 4,000,000 particles/cm^3^ between 10 and 1000 nm. Over half of these particles were nanoparticles. As a point of comparison, approximately 8.8×10^9^ particles ranging from 6–50 nm in diameter are present in the smoke from one conventional cigarette [25]. Total particle/nanoparticle doses received by EC users will vary with the number of puffs/day and remain to be determined in future studies. However, it can be estimated from our data that EC users who take 100 puffs/day on the brand used in this study will inhale approximately 10^8^ particles <1000 nm, and we know from TEM analysis that some of these nanoparticles will be heavy metals.

Nanoparticles tend to penetrate deep into the respiratory system and reach the alveolar sacs [26], [27]. The brand of EC used in our study required a high air flow rate to produce aerosol, which would further enhance deep penetration of nanoparticles into the respiratory system [27]. Metal particles/nanoparticles can produce adverse effects *in vitro* and *in vivo* [28], [29], and nanoparticles can be transported *in vivo* to other organs, including the liver, kidney, heart, and brain [30], [31]. Chromium, nickel and tin nanoparticles were found in EC aerosol. Cobalt chromium and chromium oxide nanoparticles damaged cultured cells [32], [33], and long-term inhalation of nickel hydroxide nanoparticles by mice caused oxidative stress and inflammation in lung and cardiac tissues [34]. While tin nanoparticles, per se, have not been studied in human lungs, tin dust from a Chinese mine was cytotoxic, induced release of reactive oxygen species from alveolar macrophages *in vitro* [35], and induced malignant transformation of cultured bronchial epithelial cells [36]. Inhalation of tin dust has also been reported to cause stannosis in humans [37].

A total of 22 elements were identified in EC aerosol, and three of these elements (lead, nickel, and chromium) appear on the FDA's “harmful and potentially harmful chemicals” list [38]. Lead and chromium concentrations in EC aerosols were within the range of conventional cigarettes, while nickel was about 2–100 times higher in concentration in EC aerosol than in Marlboro brand cigarettes (Table 1). Adverse health effects in the respiratory and nervous systems can be produced by many of the elements in Table 1, and many of the respiratory and ocular symptoms caused by these elements have been reported by EC users in the Health and Safety Forum on the Electronic Cigarette Forum website (http://www.e-cigarette-forum.com/forum/health-safety-e-smoking/). Although Table 1 was constructed to emphasize the effects of the elements found in aerosol on the respiratory system, other systems, such as the cardiovascular and reproductive systems, can be affected by most of the elements in EC aerosol. EC consumers should be aware of the metal and silicate particles in EC aerosol and the potential health risks associated with their inhalation.

Our studies are based on one brand of EC purchased on multiple occasions over a two year period. It will be important in future studies to determine if other brands of EC produce aerosol with similar levels of metals and silicates and if the metals, silicates and nanoparticles in EC aerosol present long-term health risks to users and exposed bystanders.

### Conclusion

Cartomizer aerosol from a leading manufacturer of EC contained metals, silicate beads, and nanoparticles. Poor solder joints appear to have contributed to the presence of tin in the aerosol. In cytotoxicity tests, cartomizer fluid containing tin particles inhibited attachment and survival of hPF. Other metals likely came from the wires (copper, nickel, silver) and other metal components used in the cartomizers, while silicate particles appeared to come from the fiberglass wicks. While the outer fibers filtered out many of the tin particles, significant amounts of tin, other metals, and silicate beads escaped into the aerosol and would result in human exposure, in some cases probably greater than a conventional cigarette user would experience. These data should be helpful to individuals who are concerned with the health risks associated with EC use, to health care personnel advising EC users, and to policy makers. The presence of silicate particles and metal elements in EC aerosol may help guide manufacturers in selection of materials for use in EC products and in their quality control procedures.

## Methods

### Cartomizers, culture medium, and reagents

EC kits and packs from a well-known manufacturer were purchased from local retailers or on the Internet and stored at room temperature. Each pack contained three replacement cartomizers. All cartomizers tested contained 0 mg of nicotine and represented the same product from the same company. Eight different packs were acquired over a 2-year period in four different purchases. hPF and human fibroblast medium bullet kit were purchased from ScienCell (Carlsbad, CA). Trypsin (Invitrogen, Grand Island, NY) was diluted to 0.05% with Dulbecco's phosphate buffered saline without Ca^2+^ or Mg^2+^ (Invitrogen, Grand Island, NY) and stored at 4°C. Fetal bovine serum (Invitrogen, Grand Island, NY) was heat inactivated, aliquoted, and stored frozen at −20°C. Poly-L-lysine (ScienCell, Carlsbad, CA) was used to coat tissue culture dishes/plates before plating cells.

### Cartomizer dissection

The white plug in the end of the mouthpiece and associated clear ring were removed, revealing cartomizer fluid and outer fibers. The latter were gently removed using forceps, exposing the wires and inner fibers. After unwrapping the inner fibers from the wires, the air tube assembly with the associated wires was removed with forceps. For each cartomizer, the following were recorded: the pack inventory code, cartomizer number, fiber type centrifuged, amount of fluid recovered, fluid color, condition of the solder and wick, evidence of electrophoresis, and burning of the inner fibers. Cartomizer dissections were photographed using a Canon SLR digital camera, and individual components were imaged with a Nikon SMZ 745 stereoscope and Nikon Eclipse Inverted microscope.

### Fluid separation and pellet weight analysis

The inner and outer fibers were centrifuged either together or separately in MinElute Spin Columns (Qiagen, Valencia, CA) at 14,000 revolutions per minute for 4–6 minutes to remove fluid from the fibers. The volume of recovered fluid in each centrifuge tube was estimated using a volumetric pipette. The wet weight and dry weight of pellets formed during centrifugation were determined.

### Preparing cartomizer liquids for cytotoxicity assays

Two solutions were tested for cytotoxicity. Supernatant from a cartomizer that had been centrifuged once using a Mini MicroElute Spin column was compared to supernatant from the same cartomizer that had some of the pellet material resuspended in it. The pellet in this experiment was produced by centrifuging both the outer and inner fibers. These solutions were prepared using sterile technique, aliquoted into Eppendorf tubes, and stored at room temperature until used.

### hPF culture

hPF (ScienCell, Carlsbad, CA) were cultured using the ScienCell protocol in complete fibroblast medium containing 2% fetal bovine serum, 1% fibroblast growth serum, and 1% penicillin/streptomycin. hPF were grown on poly-L-lysine (15 µL/10 mL) coated Nunc T-25 flasks (Thermo Scientific, Rochester, NY), which were prepared the day before use. Cells were examined daily for normal morphology, and medium was changed every other day. hPF were cultured in 5% carbon dioxide at 37°C and 95% relative humidity until 85% confluent, at which time they were used for cytotoxicity testing. Stock 0.25% trypsin-EDTA (Gibco by Life Technologies, Grand Island, NY) was diluted in Ca^2+^/Mg^2+^-free Dulbecco's phosphate buffered saline to form a working concentration of 0.01%, which was used to remove cells from the poly-L-lysine coated surfaces for subculturing and experiments.

### Evaluating cytotoxicity with live cell imaging and the MTT assay

Live cell imaging of hPF treated with cartomizer fluid with or without particles was performed in a BioStation CT (Nikon, Melville, NY) for 48 hours. 120,000 cells/well were plated in 24-well tissue culture plates (Fisher, Chino, CA) in the presence of cartomizer solution with or without pellet particles (0.01% or 1%) or in culture medium alone. Images were taken at 5 regions in each well once every hour for 48 hours. Evaluation of hPF attachment in control and treated groups was done by counting the percentage of attached cells in five images from the 8^th^ hour of incubation by which time attachment was complete. Confluency was compared in control and treated groups with CL Quant software using five images from the 32 hour time point.

To evaluate cytotoxicity with the MTT assay, 20,000 hPF/well were plated on poly-L-lysine coated 96-well plates (Fisher, Chino, CA). After 24 hours, cartomizer fluids with or without particles were added at concentrations 0.0%, 0.1, or 1.0%. Cells were incubated with cartomizer fluids for 48 hours, then incubated with MTT (20 µL of 5 mg/mL) (Sigma, St. Louis, MO.) for 2 hours at 37°C. Solutions were removed from each well, and 100 µL of dimethylsulfoxide (Fisher, Chino, CA) was added, and the absorbance of each well at 570 nm was determined using an Epoch (Biotek, Winooski, VT) microplate reader.

### Preparing liquid and solid samples for scanning electron microscope (SEM) and x-ray microanalysis

Pin stubs were rinsed with acetone, sonicated for 1 hour in acetone, rinsed in acetone, and dried in a glass Petri dish. To prepare cartomizer fluid, 20 µL of supernatant from centrifuge tubes were spread over the surface of pin stubs, then were fully dried. In some cases, fluid on fibers from an area of interest was directly spread on pin stubs, then dried. Pellets from centrifuge tubes were lifted with a spatula and smeared onto pin stubs. Solid samples (fibers, wires) were attached to pin stubs covered with carbon tape and examined without coating.

### Preparing aerosol and room air samples for SEM and TEM

Aerosol was generated on a smoking machine as described previously [6], [18]. Tubing was cleaned and dried before use. Aerosol was captured inside a loosely covered 200 mL beaker containing either a pin stub for SEM or formvar coated copper grid for TEM. Either 15 puffs (TEM) or 60 puffs (SEM) were taken using a fully charged battery and fresh cartomizer. Each puff lasted 4.3 seconds [39]. The pin stubs and copper grids were dried before examination in the SEM or TEM. Samples of room air were prepared the same way except room air, not aerosol, was pumped into the beaker containing either a pin stub or formvar coated grid.

### SEM and TEM x-ray microanalysis

Sample ultrastructure, size, and elemental composition were examined in either a FEI XL30 FEG SEM using backscatter and/or secondary electron imaging modes or using a FEI CM300 TEM. Elemental analysis of samples was done by energy dispersive x-ray spectroscopy (EDS) in the XL30 SEM fitted with EDAX® Genesis system and 10 mm^2^ Si(Li) detector and in the CM3000 TEM fitted with EDAX® Genesis system and 30 mm^2^ Si(Li) detector.

### Particle counting and sizing

The smoking machine was connected to a TSI 3772 Condensation Particle Counter (TSI, Shoreview, MN), and the number of particles/puff was quantified during puffing of an EC. To determine particle size, aerosol produced on the smoking machine was collected in a glass jar, then analyzed sequentially using a TSI 3080 Scanning Mobility Particle Sizer and the Condensation Particle Counter. Three trials were performed for single puffs of 4.3 second durations. Room air was analyzed in a similar manner.

### Preparing aerosol and room air samples for ICP-OES

Aerosol was produced using the smoking machine as described previously [6]. Tygon tubing was cleaned and dried before use. Aerosol was captured in a 500 mL round bottom flask covered with Parafilm. A small glass capillary served as an exhaust. Aerosol solutions were prepared from three fresh cartomizers. For each cartomizers, 60 puffs (4.3 seconds each), and aerosol was allowed to fully dissolve in a solution of 10% nitric acid, 3% hydrochloric acid, and 87% deionized water before the next puff was added to the flask. Room air was prepared in a similar fashion. All samples were stored in 15 mL conical vials. ICP-OES analysis was used to quantify the concentrations of elements in the aerosol using an Optima 7300 PV (Perkin-Elmer, Waltham, MA.).

## Supporting Information

Figure S1 **EDS microanalysis of cartomizer components.** SEM micrographs (inserts) and EDS spectra of (A) the wick, (B) the mouthpiece, (C) the air tube, and (D) the silicon gasket.
(TIF)
