# Supplementary figures and images for: Metal and Silicate Particles Including Nanoparticles Are Present in Electronic Cigarette Cartomizer Fluid and Aerosol
Source: PLoS One. 2013 Mar 20;8(3):e57987. doi: 10.1371/journal.pone.0057987 (PMC3603976; doi:10.1371/journal.pone.0057987)

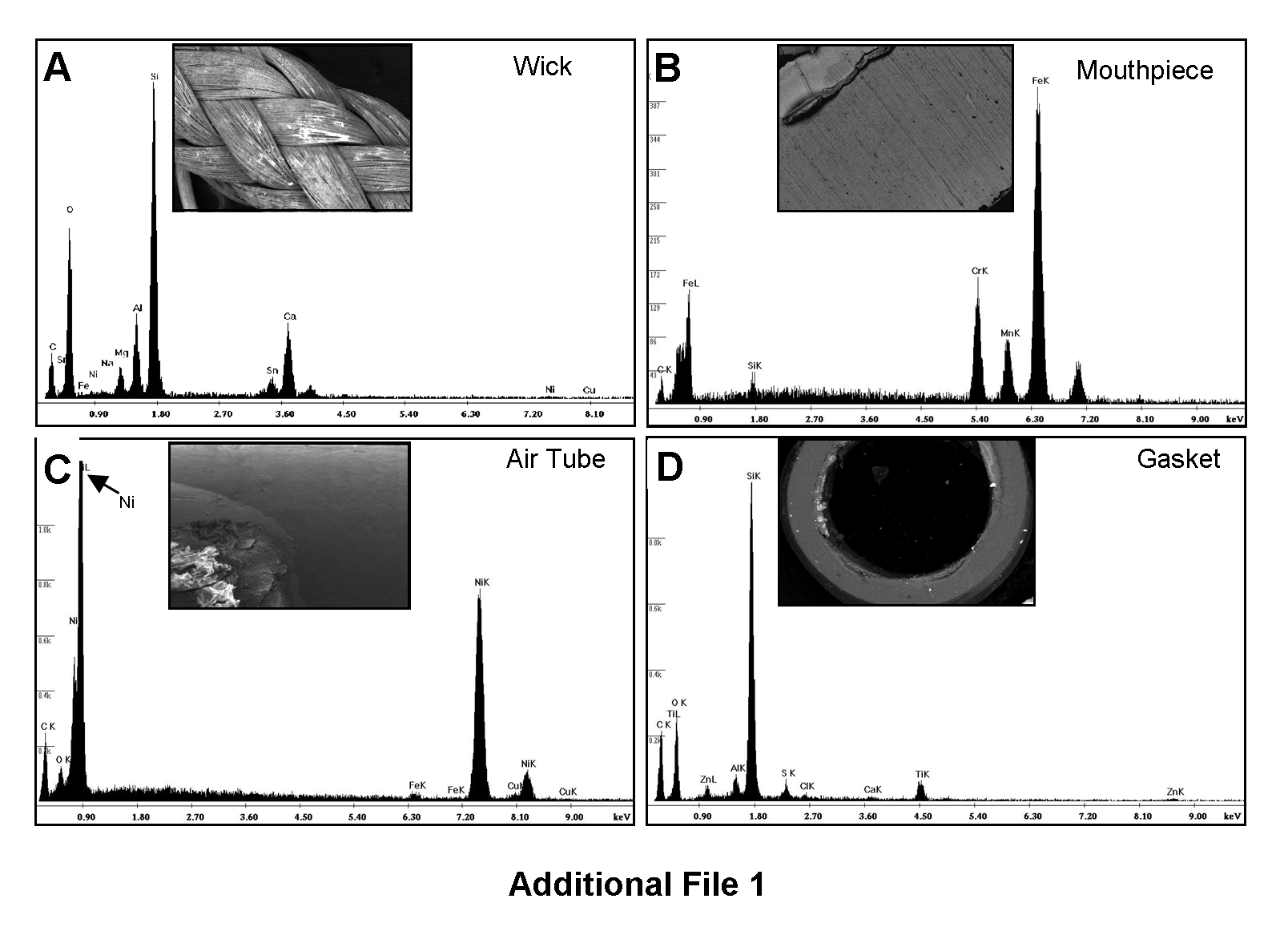

Supplement: Figure S1 — EDS microanalysis of cartomizer components. SEM micrographs (inserts) and EDS spectra of (A) the wick, (B) the mouthpiece, (C) the air tube, and (D) the silicon gasket. (TIF) [file pone.0057987.s001.tif]
